# Mexico’s First Heart Transplant in a Patient with Becker Muscular Dystrophy Reveals an Overlooked Cause of Idiopathic Dilated Cardiomyopathy

**DOI:** 10.3390/muscles5030057

**Published:** 2026-08-11

**Authors:** Norma Alejandra Vázquez-Cárdenas, Silvia García, Martha Eunice Rodríguez Arellano, Benjamín Gómez-Díaz, Delia Carolina López Vargas, Luz Berenice López-Hernández

**Affiliations:** 1 Life Cycle Department, Universidad Autónoma de Guadalajara, Av. Patria 1201, Zapopan 45129, Mexico; alejandra.vazquez@edu.uag.mx; 2 Instituto de Seguridad y Servicios Sociales de los Trabajadores del Estado, Mexico City 03104, Mexico; rolasil@yahoo.com.mx (S.G.); marthaeunicer@hotmail.com (M.E.R.A.); 3 Instituto Nacional de Rehabilitación Luis Guillermo Ibarra Ibarra, Mexico City 14389, Mexico; bgodiaz@gmail.com; 4 Asociación de Distrofia Muscular de Occidente A.C., Guadalajara 44380, Mexico; carolina.lopez.vargas96@gmail.com

**Keywords:** heart failure, dystrophin, Becker muscular dystrophy, dilated cardiomyopathy, heart transplantation, MLPA

## Abstract

Background: Pathogenic variants in the DMD gene are a frequently overlooked cause of dilated cardiomyopathy (DCM) in idiopathic cases, especially when skeletal myopathy is mild or seemingly absent. Becker muscular dystrophy (BMD) and X-linked dilated cardiomyopathy are at opposite ends of this clinical spectrum but result from pathogenic variants in the same gene. Cardiomyopathy is a major cause of premature death in muscular dystrophies. Case presentation: We describe a 37-year-old man who underwent orthotopic heart transplantation for presumed idiopathic DCM with refractory heart failure. He had a six-year history of progressive proximal weakness, pseudohypertrophy of the calf, a positive Gowers sign, and markedly elevated creatine kinase (6638 IU/L). Multiplex ligation-dependent probe amplification (MLPA) revealed an in-frame deletion of exons 45–47 of DMD [c. (6438+1_6439-1)_(6912+1_6913-1) del; NM_004006.2], confirming BMD. Immunofluorescence showed reduced and patchy dystrophin expression. Conclusions: To our knowledge, this is the first documented heart transplant in a patient with BMD in Mexico. This case demonstrates the value of considering muscular dystrophy in the differential diagnosis of apparently idiopathic dilated cardiomyopathy (DCM), a step that opens the door to accurate diagnosis, carrier screening, and genetic counseling, and supports the view that heart transplantation is a viable option in carefully selected patients with muscular dystrophy cardiomyopathy.

## 1. Introduction

Dystrophinopathies are a group of X-linked neuromuscular disorders caused by pathogenic variants in the DMD gene, which encodes dystrophin. This protein is part of the cytoskeleton and plays an essential role in maintaining the structural integrity of striated muscle by connecting the sarcolemma to the extracellular matrix, thus protecting muscle fibers from the mechanical stress generated during each contraction cycle. When dystrophin is absent or produced in a truncated form, the cell membrane becomes more vulnerable to damage, leading to repetitive myocyte injury, inflammation, fibrosis, and progressive muscle degeneration that primarily affects skeletal and cardiac muscle [1,2,3].

Within this clinical spectrum, two main neuromuscular disorders are distinguished. Duchenne muscular dystrophy (DMD; MIM #310200) represents the most severe form of the disease, with onset in early childhood and rapid progression to loss of muscle function. In contrast, Becker muscular dystrophy (BMD; MIM #300376) typically manifests at later ages and exhibits a much more variable course, since frame-preserving genetic variants allow for the synthesis of partial amounts of functional dystrophin [4].

The relationship between genotype and phenotype is largely explained by the so-called reading frame rule. According to this principle, frame-preserving deletions generally result in the milder phenotype of BMD, while frame-shifted deletions usually lead to the more severe form of DMD. This rule holds true in approximately 90% of patients, although there are well-documented exceptions, particularly in variants affecting the rod domain of dystrophin [1,5].

The clinical spectrum of dystrophinopathies, however, extends beyond these two entities. BMD can remain undiagnosed for years, particularly when cardiomyopathy is the predominant manifestation and masks skeletal muscle weakness. At the opposite end of the spectrum is X-linked dilated cardiomyopathy (XL-DCM; MIM #302045), characterized by virtually isolated cardiac involvement, in which subclinical skeletal muscle dysfunction may silently precede the development of overt heart failure.

The clinical significance of this phenotype has been supported by recent studies. In a large European multicenter cohort, carriers of DMD variants without significant skeletal myopathy showed incomplete penetrance of dilated cardiomyopathy (45% in individuals over 40 years of age), although with an elevated risk of major adverse cardiovascular events. Notably, the prognosis was determined primarily by the development of dilated cardiomyopathy and not by the severity of skeletal muscle involvement [6]. Similarly, systematic analysis of structural variants of CNVs in cohorts of patients referred for genetic evaluation of dilated cardiomyopathy identified DMD as the gene most frequently affected by pathogenic variants, highlighting the relevance of dystrophinopathies in cases previously considered idiopathic [7].

These observations underscore that cardiac disease is an integral manifestation of dystrophinopathies rather than simply a late consequence of skeletal muscle degeneration. Indeed, cardiomyopathy is among the leading causes of death in affected individuals and accounts for a substantial proportion of their premature mortality [2].

Cardiac damage begins at the cellular level, where the absence or deficiency of dystrophin compromises the structural stability of cardiomyocytes. Repeated mechanical stress leads to myocyte necrosis, progressive fibrosis, and fibro-fatty replacement, changes that predominantly affect the free wall of the left ventricle. Over time, these pathological alterations promote ventricular remodeling, resulting in chamber dilation and systolic dysfunction that eventually progress to refractory heart failure. At the same time, abnormalities of the cardiac conduction system and ventricular arrhythmias markedly increase the risk of sudden cardiac death [8,9]. Disturbances in intracellular calcium homeostasis together with mitochondrial dysfunction further exacerbate myocardial injury and disease progression [3].

Although cardiomyopathy develops in both Duchenne and Becker muscular dystrophies, its clinical presentation differs considerably. In Duchenne muscular dystrophy, cardiac involvement typically evolves in parallel with progressive multisystem deterioration and often remains clinically silent because severe skeletal muscle weakness dominates the clinical picture until advanced stages. By contrast, in Becker muscular dystrophy and X-linked dilated cardiomyopathy (XL-DCM), cardiac manifestations may become the predominant feature of the disease, with dilated cardiomyopathy representing the principal source of morbidity despite relatively mild skeletal muscle impairment [10].

Evidence guiding the management of heart failure specifically associated with BMD and XL-DCM is still limited. Nevertheless, current experience suggests that guideline-directed medical therapy, together with device-based interventions when appropriate, can improve symptoms and quality of life in selected patients [9]. For individuals who progress to end-stage heart failure despite optimal treatment, heart transplantation (HTx) remains the only definitive therapeutic option. Encouragingly, contemporary studies have shown that survival following transplantation in patients with dystrophinopathies is comparable to that observed in recipients transplanted for other forms of non-ischemic cardiomyopathy [11,12,13] (Table 1).

Here, we describe what is, to the best of our knowledge, the first reported case of heart transplantation in a Mexican patient with Becker muscular dystrophy. This case also highlights the clinical importance of considering an underlying dystrophinopathy in patients presenting with apparently idiopathic dilated cardiomyopathy, as establishing the correct diagnosis has significant implications for patient management, family counseling, and genetic risk assessment.

## 2. Case Description

A 37-year-old Mexican Mestizo man was referred for evaluation because of progressive proximal muscle weakness, increasing difficulty walking, and worsening shortness of breath. His early motor milestones were normal, and he achieved independent walking at 13 months of age. Throughout childhood, he experienced intermittent calf pain and gradual enlargement of both calves, which he attributed to physical conditioning. During adolescence, he began to experience frequent falls and exercise intolerance; however, these symptoms were never investigated. Functional limitations became evident at approximately 26 years of age, when he noticed increasing difficulty climbing stairs, walking long distances, and rising from low chairs. Despite these progressive symptoms, he remained physically active and continued to play amateur soccer until worsening cardiac function ultimately limited his exercise capacity.

The patient was married and had one daughter. He reported no family history of neuromuscular disorders or cardiovascular disease, and there was no previous record of neurological evaluation, cardiac disease, or long-term medical treatment before his current presentation.

The initial clue emerged during a hypercholesterolemia screening program at the Clínica de Lípidos y Cardiología, UMAE Hospital de Especialidades. Routine laboratory testing revealed a markedly elevated serum creatine phosphokinase (CPK) concentration of 2220 IU/L (reference range: 20–180 IU/L), prompting referral to a clinical geneticist (A.V.C.). Physical examination demonstrated prominent bilateral calf pseudohypertrophy, a waddling gait, and a positive Gowers’ sign, findings consistent with proximal lower-limb muscle weakness. Weakness was also evident in the proximal muscles of both the upper and lower extremities, making it difficult for the patient to stand from a seated position, climb stairs, or lift his arms above shoulder height. Generalized hypotonia was present at rest.

Repeat laboratory evaluation showed a serum creatine kinase (CK) level of 6638 IU/L, approximately 37 times the upper limit of normal (reference range: 20–180 IU/L). As muscle weakness progressed, exertional dyspnea evolved into orthopnea, prompting comprehensive cardiac evaluation. Transthoracic echocardiography demonstrated left ventricular dilation accompanied by severe global hypokinesia and markedly reduced systolic function, although the exact ejection fraction was not documented. Electromyography supported the presence of a primary myopathic process.

The patient was initially diagnosed with idiopathic dilated cardiomyopathy complicated by advanced heart failure. Despite receiving optimized guideline-directed medical therapy—including an angiotensin-converting enzyme inhibitor, a beta-blocker, and a loop diuretic at the highest tolerated doses—his clinical condition continued to deteriorate. Consequently, orthotopic heart transplantation was recommended and successfully performed on February 20, 2009, at Unidad Médica de Alta Especialidad No. 145, Centro Médico Nacional de Occidente, Instituto Mexicano del Seguro Social.

## 3. Results

During the pre-transplant evaluation, the combination of male sex, bilateral calf pseudohypertrophy, progressive proximal muscle weakness, and markedly elevated CK levels strongly suggested an underlying dystrophinopathy. Initial molecular testing using multiplex PCR targeting 14 exons of the DMD gene (Dp427m exons 4, 8, 12, 13, 17, 19, 43–45, 48, and 50–52) identified a deletion involving exon 45. Because an isolated exon 45 deletion is predicted to disrupt the reading frame and would generally be expected to produce the more severe Duchenne phenotype, multiplex ligation-dependent probe amplification (MLPA) was subsequently performed for confirmation.

A left deltoid muscle biopsy was obtained. Histological evaluation (hematoxylin–eosin, Gomori trichrome) revealed fiber size variability, with rare small rounded fibers and a mild increase in inter- and intrafascicular adipose tissue; no inflammatory infiltrates or abnormal aggregates were identified. Oxidative enzyme histochemistry (NADH-TR) and additional histochemical stains (PAS, Oil Red O, COX) were unremarkable. These findings were compatible with a chronic myopathic process.

**Figure 1:**
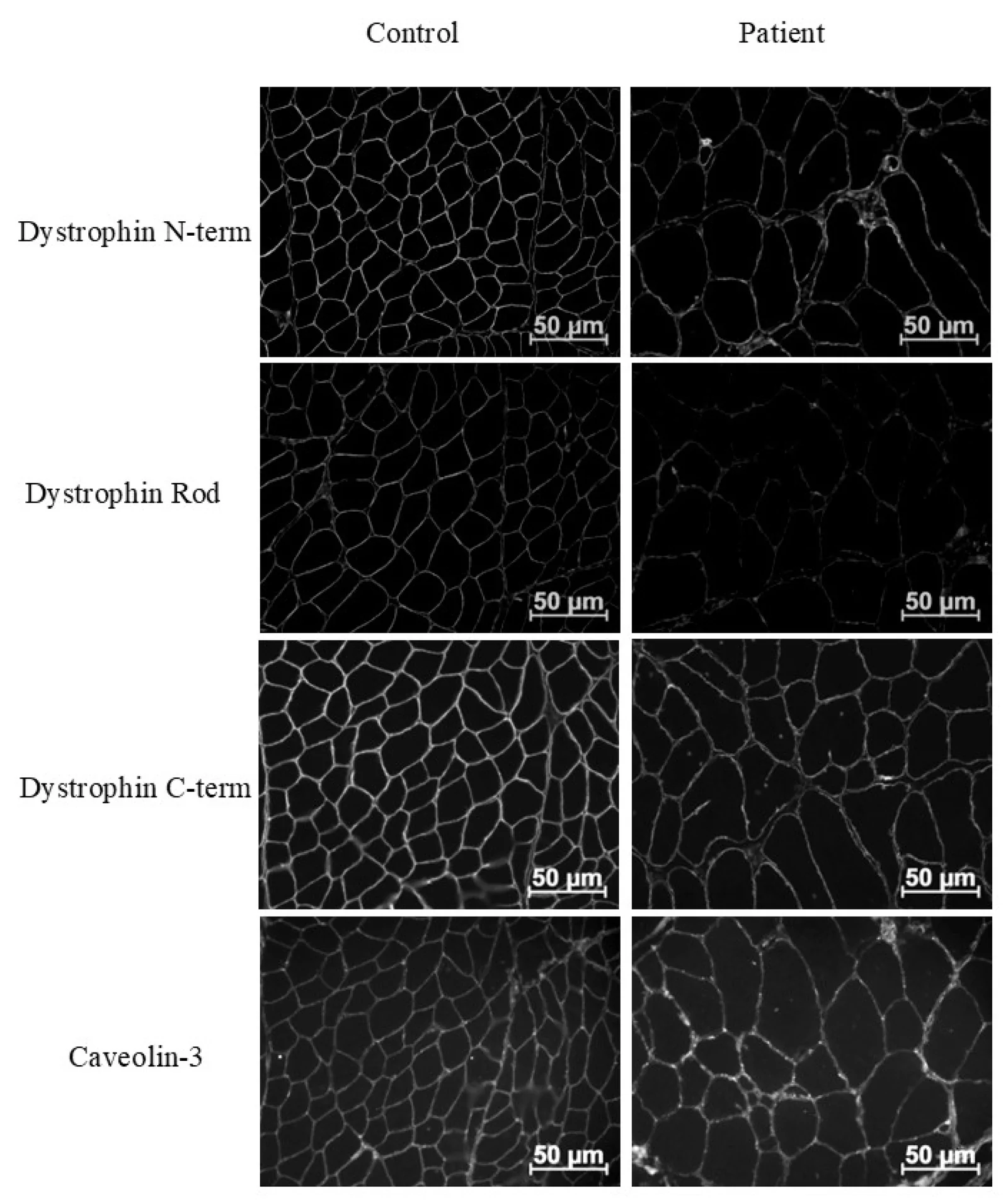
Muscle biopsy immunofluorescence. Compared with control, the patient’s biopsy shows diminished dystrophin expression in the rod domain. (Additional figures in color as Supplementary Material).

Immunofluorescence analysis of the same biopsy, performed against three dystrophin domains, demonstrated a domain-dependent pattern of protein loss: staining was most severely reduced for the rod-domain epitope (NCL-DYS1), with near-complete loss of sarcolemmal signal in most fibers; N-terminal staining (NCL-DYS3) was moderately reduced and discontinuous; and C-terminal staining (NCL-DYS2) was partially preserved, with heterogeneous fiber-to-fiber intensity—some fibers retaining near-normal signal and others markedly reduced (Figure 1). Caveolin-3 staining, used as an internal control for sarcolemmal membrane integrity, showed a patchier, redistributed pattern compared with the uniform membrane signal in the control, a change that has been described as a secondary consequence of dystrophin–glycoprotein complex disruption rather than a primary membrane defect. This mosaic, domain-dependent pattern—most pronounced at the rod domain with partial preservation of the C-terminus—is consistent with an in-frame deletion permitting synthesis of a truncated, partially functional dystrophin protein, in keeping with the molecular diagnosis of Becker muscular dystrophy. Unfortunately, no Western blot was available due to the lack of biological specimen after the above-mentioned analyses. Control muscle tissue was obtained from residual samples of orthopedic surgical procedures (e.g., back/shoulder surgeries) in patients without neuromuscular disorders.

Subsequent MLPA analysis of all 79 DMD exons (SALSA P035/P036) confirmed a deletion spanning exons 45–47 [c.(6438+1_6439-1)_(6912+1_6913-1)del; DMD, NM_004006.2]. This in-frame deletion is predicted to remove amino acids Glu2146 to Val2304 within the central rod domain of dystrophin—consistent with the observed BMD phenotype and with the reading-frame rule [1,19].

Two years after transplantation, the patient had stable graft function and no evidence of rejection; his skeletal-muscle function, however, did not appreciably improve. He took part in a research study approved by the local research and ethics committees (reference 094.2013/ISSSTE, Centro Médico Nacional “20 de Noviembre”), and written informed consent was obtained. Genetic counseling was offered to the family, including a frank discussion of the daughter’s status as an obligate carrier. Ongoing cardiac surveillance was recommended, given reports of rapidly progressive heart failure in female BMD carriers even without skeletal-muscle symptoms [20,21]. The patient stayed in contact via social media from 2009 to 2015; after that, no further clinical follow-up was possible.

## 4. Discussion

Heart transplantation is a critical intervention for end-stage DCM in patients with dystrophinopathies, as this case illustrates. It underscores the life-saving potential of HTx while drawing attention to the often underrecognized neuromuscular features of dystrophinopathies, which can complicate perioperative management. Converging evidence indicates that BMD and XL-DCM account for a meaningful share of cases first labelled idiopathic DCM: structural variants in DMD are the most frequently identified copy-number cause in large, genetically evaluated DCM cohorts [7], and dystrophin-associated DCM occurs even without severe skeletal myopathy [6]. Together these observations argue for heightened clinical suspicion, thorough phenotyping, and early genetic testing to sharpen diagnosis and counseling [11].

**Figure 2:**
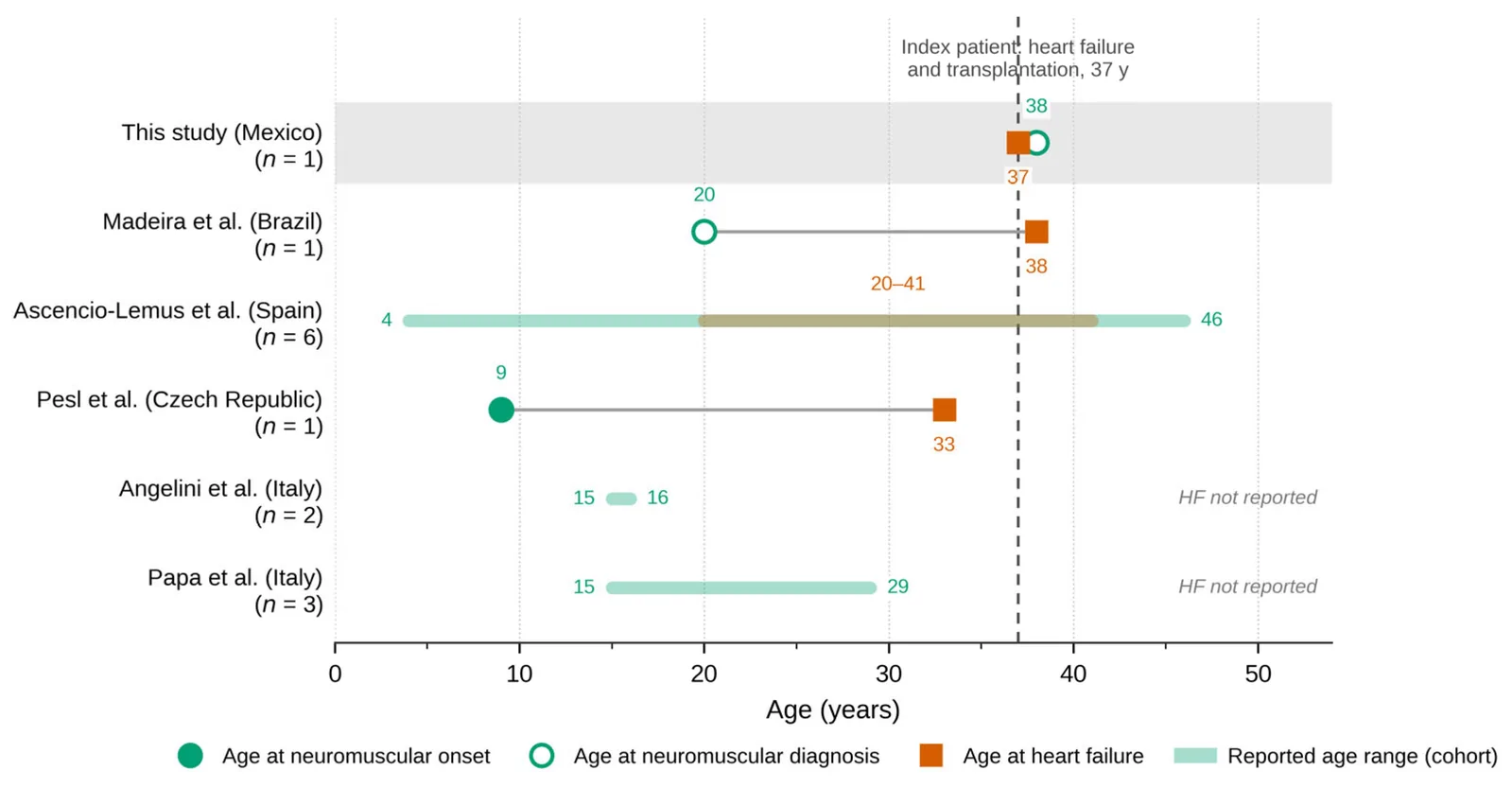
Age at neuromuscular onset or diagnosis (green) versus age at heart failure (orange) in published Becker muscular dystrophy cases with cardiac involvement [16,23,24,25,26]. Translucent bars denote reported cohort ranges, and connecting lines link paired values within a study. The index patient (highlighted) was diagnosed with neuromuscular disease only at age 38, after heart failure had necessitated transplantation at 37 (dashed line).

Before the diagnosis of BMD, the patient’s presumed idiopathic DCM had already undergone standard exclusion of common secondary causes as part of his pre-transplant cardiac evaluation, including coronary angiography, which excluded significant coronary artery disease, and clinical history, which showed no evidence of alcohol use disorder. Myocarditis was not formally excluded, as endomyocardial biopsy, cardiac MRI with late gadolinium enhancement, and viral serology were not performed as part of the original 2009 evaluation; this is acknowledged as a limitation of the retrospective work-up. Nonetheless, the combination of bilateral calf pseudohypertrophy, proximal weakness, a positive Gowers’ sign, and a CK level approximately 37-fold above the upper limit of normal are not features of ischemic, viral, or alcohol-related cardiomyopathy, and their presence—in retrospect—should have prompted earlier consideration of an underlying myopathic/dystrophinopathic process regardless of the cardiac work-up performed. In our patient, calf hypertrophy, proximal weakness, a positive Gowers’ sign, and a CK elevated roughly 35-fold above the upper reference limit pointed firmly toward a dystrophinopathy, which preoperative multiplex PCR and subsequent MLPA confirmed. The in-frame exon 45–47 deletion lies within the central rod domain, a region where deletions are generally associated with later-onset cardiomyopathy than amino-terminal deletions [22]. Even so, he needed transplantation at 37—within the range reported for rod-domain genotypes and broadly in line with the cohort summarized in Table 1 (Figure 2).

His history of sustained, vigorous exercise raises the possibility that exercise-related cardiac loading contributed to disease progression, though that cannot be established from a single case. The hypothesis is at least biologically plausible: in dystrophin-deficient mice, selectively repairing skeletal muscle increased voluntary activity and paradoxically intensified cardiac injury and dilated cardiomyopathy, implicating activity-driven mechanical stress on a vulnerable myocardium [27]. Impaired neuronal nitric oxide synthase–mediated sympatholysis in dystrophic muscle may further compromise myocardial perfusion during exertion [28]. These findings point to a real need for evidence-based activity guidance in dystrophinopathies, particularly for those at risk of rapid cardiac decline—while acknowledging that exercise effects in dystrophic muscle are dose-dependent and not uniformly harmful, so firm recommendations await prospective study.

Recognizing dystrophinopathy carries direct consequences for the proband’s relatives. The patient’s daughter is an obligate carrier, and female carriers can develop DCM and arrhythmia—detectable by cardiac magnetic resonance and exercise testing—even without skeletal-muscle symptoms [20,21]. Cascade genetic testing and longitudinal cardiac surveillance of carriers are therefore warranted.

Disease modeling in dystrophinopathies remains difficult. Approaches based on skeletal-muscle biopsy or conventional animal models often fail to reproduce human cardiac pathophysiology, partly because human cardiac tissue is scarce and standard mdx mice do not develop early dilated cardiomyopathy [29]. Induced pluripotent stem cell-derived cardiomyocytes (iPSC-CMs) offer a patient-specific, genetically faithful alternative that recapitulates aspects of human cardiac dystrophinopathy [25,30]. Current iPSC-CM models are still limited by cellular immaturity and incomplete reproduction of the native tissue microenvironment; next-generation engineered-tissue platforms aim to mature cardiomyocytes further, simulate tissue-level interactions, and enable higher-throughput drug screening. This case underscores the need for prospective, multi-institutional studies systematically evaluating skeletal muscle involvement—including CK levels and targeted genetic testing—in patients presenting with apparently idiopathic DCM, particularly candidates being evaluated for heart transplantation. A structured screening protocol incorporating routine CK measurement and, where elevated, cascade genetic testing for DMD variants, applied prospectively across transplant referral centers in Mexico, would help clarify the true prevalence of unrecognized dystrophinopathy in this population and inform whether earlier diagnosis changes perioperative management or long-term outcomes.

This report has the limitations inherent to a single retrospective case. Follow-up ended in 2015, late post-transplant graft function was not fully documented, myocarditis and other inflammatory/infiltrative causes of dilated cardiomyopathy were not formally excluded via endomyocardial biopsy, cardiac MRI, or viral serology, and certain quantitative parameters were unavailable, as noted in the text. Even so, the case adds to a growing literature suggesting that—contrary to earlier reluctance—HTx outcomes in selected patients with muscular dystrophy are comparable to those in matched recipients with non-ischemic cardiomyopathy [12,13,31], a conclusion reinforced by recent series and meta-analytic data [14,15]. Recent reviews further emphasize that eligibility for advanced heart failure therapies in dystrophinopathy warrants individualized, ethically grounded, multidisciplinary evaluation, given the historically variable experience of transplant centers with this population [32]. Compared with the cohorts summarized in Table 1, this case offers several distinguishing features. Most published series report outcomes in patients whose dystrophinopathy diagnosis preceded or coincided with the decision to transplant, allowing perioperative planning around known neuromuscular risk. In our patient, by contrast, the underlying dystrophinopathy was not identified until after transplantation, when a persistently myopathic clinical picture prompted molecular testing—meaning the diagnosis of BMD only reclassified a case previously labeled as idiopathic DCM. This sequence more directly illustrates the diagnostic blind spot this report aims to highlight: that dystrophinopathy can masquerade as isolated idiopathic DCM even at the point of transplant evaluation. In addition, to our knowledge this is the first such case reported from Mexico, and one of few in the literature with documented multi-year follow-up data on both graft function and skeletal muscle status after transplantation, contributing longitudinal information largely absent from the pooled/aggregate cohorts in Table 1.

## 5. Conclusions

This case shows that BMD can present chiefly as dilated cardiomyopathy and be mistaken for idiopathic DCM until end-stage heart failure forces the issue at transplantation. To our knowledge, it is the first documented heart transplant in a patient with BMD in Mexico. Considering dystrophinopathy early in the differential diagnosis of DCM—guided by clinical examination, CK measurement, and targeted genetic testing such as MLPA—enables accurate diagnosis, carrier detection, and informed genetic counseling. While HTx remains a cornerstone of therapy for end-stage dystrophinopathic cardiomyopathy and yields favorable outcomes in selected patients, the best care is interdisciplinary, weaving together cardiologic, neuromuscular, genetic, and rehabilitative expertise, and supported by continued research to refine risk stratification and personalize management.

## Figures and Tables

**Table 1:** Post-transplantation outcomes in patients with dystrophinopathy, ordered from most to least recent.

Study	Cohort	Follow-Up	Survival	Rejection and Adverse Events
This study, 2026 Case report	N = 1 BMD with DCM	24 mo ^a^	Alive with stable graft function at 24 mo	No rejection. No graft involvement; no improvement in skeletal-muscle function after HTx; pre-HTx CK 6638 IU/L; daughter an obligate carrier.
Patil et al., 2025 [14] Meta-analysis	N = 36 (pooled) Dystrophinopathy, mixed	Variable	No difference by pre-HTx functional status (*p* > 0.05)	Rejection NR. Reduced pre- and post-HTx functional status trended toward worse survival.
Bergier et al., 2025 [15] Case series	N = 5 Muscular dystrophy with DCM; all genetically confirmed	Median 16 mo	100%	Rejection NR. Respiratory or swallowing complications in 2 patients; good functional capacity overall.
Wells et al., 2020 [12] UNOS registry	N = 81 BMD ≈ 52%, DMD ≈ 48%	1987–2016	Comparable to matched cardiomyopathy controls (*p* = 0.18); superior to unmatched controls (*p* = 0.004)	No difference vs. matched controls. VAD use 16% vs. 30% (*p* = 0.017); ventilator support 0% vs. 6%.
Ascencio-Lemus et al., 2019 [16] Single-center series	N = 6 BMD	Mean 96 mo (10–230)	Long-term survival in all 6; one non-cardiac death at 19 y	No rejection with hemodynamic compromise. Acute renal failure requiring dialysis 50%; PRES 33%; statin withdrawal 33%; progression to wheelchair in 3.
Wu et al., 2010 [13] Multicenter series	N = 29 BMD 52%, DMD 48%	Up to >10 y	1-year 89%; 5-year 83% (vs. NICM, *p* = 0.5)	No difference vs. NICM. CAV and infection rates comparable to controls.
Ruiz-Cano et al., 2003 [17] Case series	N = 3 BMD	Mean 57.4 mo (13–128)	100%	Rejection NR. No graft dysfunction; successful rehabilitation in all.
Casazza et al., 1988 [18] Case report	N = 1 BMD	2 y	Alive at 2 y	Rejection NR. No graft failure; neuromuscular disability unchanged.

^a^ Clinically documented follow-up. Non-clinical contact with the patient continued until 2015. BMD, Becker muscular dystrophy; CAV, cardiac allograft vasculopathy; CK, creatine kinase; DCM, dilated cardiomyopathy; DMD, Duchenne muscular dystrophy; HTx, heart transplantation; NICM, non-ischemic cardiomyopathy; NR, not reported; PRES, posterior reversible encephalopathy syndrome; UNOS, United Network for Organ Sharing; VAD, ventricular assist device.

## Data Availability

De-identified data supporting the findings of this study are available from the corresponding author upon reasonable request.

## Supplementary Materials

The following supporting information can be downloaded at: https://www.mdpi.com/article/10.3390/muscles5030057/s1, Figure S1: three domains; Figure S2: NCL immunostaining.

## Abbreviations

Abbreviations: BMD, Becker muscular dystrophy; CAV, cardiac allograft vasculopathy; CK, creatine kinase; CM, cardiomyopathy; CNI, calcineurin inhibitor; DCM, dilated cardiomyopathy; DMD, Duchenne muscular dystrophy; HTx, heart transplantation; MD, muscular dystrophy; MLPA, multiplex ligation-dependent probe amplification; NICM, non-ischemic cardiomyopathy; NR, not reported; PRES, posterior reversible encephalopathy syndrome; VAD, ventricular assist device; XL-DCM, X-linked dilated cardiomyopathy.
